# Supplementary material for: Phytohormones as Regulators of Mitochondrial Gene Expression in Arabidopsis thaliana
Source: Int J Mol Sci. 2023 Nov 29;24(23):16924. doi: 10.3390/ijms242316924 (PMC10707152; doi:10.3390/ijms242316924)
Supplement: Supplementary file 1 [file ijms-24-16924-s001.zip › ijms-2725548-supplementary.pdf]

SUPPLEMENTARY DATA

Phytohormones as Regulators of Mitochondrial Gene Expression in *Arabidopsis thaliana*

Ivan A. Bychkov [1], Elena S. Pojidaeva [1], Anastasia S. Doroshenko [1], Vladimir A. Khripach [2], Natalia V. Kudryakova [1,]* and Victor V. Kusnetsov [1,]*

[1] Timiryazev Institute of Plant Physiology, Russian Academy of Sciences, Moscow 127276, Russia

[2] Institute of Bioorganic Chemistry, National Academy of Sciences of Belarus, 220072 Minsk, Belarus

* Correspondence: nvkudryakova@mail.ru (N.V.K.); vkusnetsov2001@mail.ru (V.V.K.)

Table S1. Effects of hormone treatment on relative expression values of marker genes involved in plant responses to exogeneous hormones. WT plants were treated with solutions of hormones or an equal aliquot of ethanol for 3h. The compounds assayed included abscisic acid (ABA, 5×10^{-5} M), gibberellic acid (GA3, 10^{-6} M), indole-3-acetic acid (IAA, auxin, 10^{-6} M), 1-aminocyclopropane-1-carboxylic acid (ACC; ethylene precursor, 10^{-5} M), *trans*-zeatin (CK, 5×10^{-6} M), brassinolide (BL, 10^{-7} M), salicylic acid (SA, 10^{-5} M) and methyl jasmonate (MJ, 5×10^{-5} M). Asterisks indicate statistically significant differences between the control variant and the treatment at $p < 0.05$ (t-test).

**Treatment**	**Transcript level**
IAA19	
WT MS	1.000 ± 0.116
WT IAA	2.815 ± 0.271*
DWF4	
WT MS	1.000 ± 0.131
WT BL	0.515 ± 0.062*
GA3	
WT MS	1.000 ± 0.144
WT GA_3	0.021 ± 0.003*
ERF1	
WT MS	1.000 ± 0.089
WT ACC	3.193± 0.325*
WT MJ	2.992 ± 0.281*
RD29	
WT MS	1.000 ± 0.104
WT ABA	4.318± 0.401*
ARR5	
WT MS	1.000 ± 0.152
WT CK	22.623± 4.016*
PR1	
WT MS	1.000 ± 0.117
WT SA	3.531± 0.366*

Table S2. Effect of phytohormone treatment on relative gene expression values of mitochondrial and nuclear genes *A. thaliana*. The wild-type and *rpotmp* mutant plants were grown on MS medium in Petri dishes at an illumination of 50 µmol $m^{-2} \cdot s^{-1}$, a temperature of 23 °C with a 16 h photoperiod. 10-day-old seedlings were treated with the solutions of hormones and collected after 3 h of exposure Total RNAs were isolated from seedlings and analyzed by relative quantitative RT-PCR using *UBQ10* and *PP2A* as internal standards. The data presented in the table are the mean values (n ≥ 3). Error bars represent SEs. Different letters denote statistically significant differences between variants within the same genotype at $p < 0.05$ (ANOVA with post hoc Tukey's multiple-comparison test); asterisks indicate statistically significant differences between the control variants of mutants and the wild type at $p < 0.05$ (t-test).

	**MS**	**CK**	**IAA**	**BL**	**GA3**	**ACC**	**ABA**	**MetJa**	**SA**
					nad6				
WT	1.000 ± 0.096^{b}	2.520 ± 0.311^{c}	2.426 ± 0.199^{c}	2.040 ± 0.217^{c}	2.624 ± 0.285^{c}	2.255 ± 0.233^{c}	2.279 ± 0.219^{c}	0.431 ± 0.051^{a}	1.739 ± 0.144^{c}
rpotmp-2	0.392 ± 0.040^{b*}	0.151 ± 0.016^{a}	0.291 ± 0.032^{b}	0.280 ± 0.025^{b}	0.313 ± 0.026^{b}	0.575 ± 0.066^{c}	0.118 ± 0.009^{a}	0.184 ± 0.022^{ab}	0.426 ± 0.051^{c}
					nad3				
WT	1.000 ± 0.141^{b}	1.398 ± 0.161^{bc}	1.388 ± 0.135^{bc}	1.427 ± 0.156^{bc}	1.547 ± 0.174^{c}	1.751 ± 0.189^{c}	1.265 ± 0.142^{b}	0.378 ± 0.044^{a}	0.913 ± 0.107^{b}
rpotmp-2	2.975 ± 0.329^{b*}	2.590 ± 0.301^{a}	3.506 ± 0.151^{b}	4.367 ± 0.514^{b}	2.835 ± 0.298^{b}	4.141 ± 0.423^{c}	2.133 ± 0.222^{a}	1.372 ± 0.142^{ab}	4.112 ± 0.399^{c}
					cob				
WT	1.000 ± 0.119^{b}	2.004 ± 0.195^{c}	1.569 ± 0.163^{c}	1.892 ± 0.232^{c}	1.849 ± 0.202^{c}	1.894 ± 0.202^{c}	1.587 ± 0.211^{c}	0.132 ± 0.255^{a}	1.363 ± 0.152^{bc}
rpotmp-2	1.401 ± 0.135^{c*}	0.971 ± 0.089^{b}	0.644 ± 0.065^{a}	1.055 ± 0.120^{b}	0.870 ± 0.096^{b}	1.838 ± 0.204^{c}	0.677 ± 0.074^{a}	0.550 ± 0.061^{a}	1.560 ± 0.125^{c}
					cox1				
WT	1.000 ± 0.125^{b}	2.227 ± 0.219^{c}	2.135 ± 0.191^{c}	1.929 ± 0.265^{c}	1.992 ± 0.213^{c}	2.128 ± 0.215^{c}	1.246 ± 0.089^{b}	0.376 ± 0.40^{a}	1.854 ± 0.177^{c}
rpotmp-2	0.651 ± 0.055^{b*}	0.515 ± 0.042^{b}	0.439 ± 0.051^{b}	0.433 ± 0.047^{b}	0.503 ± 0.050^{b}	1.082 ± 0.119^{c}	0.215 ± 0.031^{a}	0.312 ± 0.041^{a}	0.912 ± 0.083^{c}
					atp6-1				
WT	1.000 ± 0.096^{c}	1.508 ± 0.111^{d}	0.944 ± 0.066^{c}	0.942 ± 0.055^{c}	0.964 ± 0.059^{c}	1.197 ± 0.088^{c}	1.002 ± 0.049^{c}	0.091 ± 0.014^{a}	0.609 ± 0.057^{b}
rpotmp-2	1.854 ± 0.145^{c*}	1.039 ± 0.092^{b}	0.626 ± 0.052^{a}	1.633 ± 0.152^{c}	1.149 ± 0.083^{b}	2.010 ± 0.274^{c}	0.599 ± 0.051^{a}	0.660 ± 0.042^{a}	1.499 ± 0.154^{bc}
					ccMc				
WT	1.000 ± 0.121^{b}	1.861 ± 0.121^{c}	1.662 ± 0.066^{c}	2.075 ± 0.169^{c}	1.621 ± 0.078^{c}	1.442 ± 0.104^{bc}	1.674 ± 0.099^{c}	0.170 ± 0.009^{a}	1.251 ± 0.107^{b}
rpotmp-2	0.738 ±	0.423 ±	0.394 ±	0.681 ±	0.448 ±	0.537 ±	0.389 ±	0.321 ±	0.756 ±

	0.069^{b*}	0.020^{a}	0.022^{a}	0.050^{b}	0.023^{a}	0.074^{ab}	0.032^{a}	0.017^{a}	0.054^{b}
ccMFc									
WT	1.000 ± 0.114^{b}	1.760 ± 0.116^{c}	1.609 ± 0.135^{c}	1.909 ± 0.180^{c}	1.744 ± 0.145^{c}	2.087 ± 0.211^{c}	1.628 ± 0.148^{c}	0.141 ± 0.011^{a}	1.114 ± 0.096^{b}
rpotmp-2	5.965 ± 0.611^{b*}	3.872 ± 0.415^{a}	2.989 ± 0.167^{a}	6.423 ± 0.711^{b}	4.103 ± 0.325^{ab}	9.996 ± 1.006^{c}	3.316 ± 0.298^{a}	2.687 ± 0.213^{a}	6.634 ± 0.550^{b}
rps4									
WT	1.000 ± 0.121^{b}	1.520 ± 0.141^{c}	1.322 ± 0.127^{bc}	1.591 ± 0.172^{c}	0.984 ± 0.101^{b}	0.704 ± 0.082^{b}	0.868 ± 0.091^{b}	0.072 ± 0.010^{a}	0.806 ± 0.077^{b}
rpotmp-2	0.057 ± 0.007^{b*}	0.056 ± 0.004^{b}	0.070 ± 0.009^{b}	0.083 ± 0.009^{b}	0.052 ± 0.006^{b}	0.050 ± 0.004^{b}	0.023 ± 0.002^{a}	0.022 ± 0.003^{a}	0.051 ± 0.006^{b}
mttB									
WT	1.000 ± 0.113^{b}	1.922 ± 0.188^{c}	2.133 ± 0.201^{c}	2.228 ± 0.195^{c}	2.223 ± 0.195^{c}	1.983 ± 0.204^{c}	1.831 ± 0.197^{c}	0.580 ± 0.061^{a}	1.253 ± 0.135^{b}
rpotmp-2	1.463 ± 0.142^{b*}	1.334 ± 0.125^{b}	1.772 ± 0.132^{bc}	2.578 ± 0.287^{c}	1.532 ± 0.092^{b}	1.962 ± 0.177^{c}	1.440 ± 0.152^{b}	0.676 ± 0.059^{a}	2.004 ± 0.187^{c}
matR									
WT	1.000 ± 0.100^{b}	1.598 ± 0.159^{c}	1.328 ± 0.097^{bc}	1.639 ± 0.171^{c}	1.557 ± 0.149^{c}	0.818 ± 0.087^{b}	1.507 ± 0.119^{c}	0.140 ± 0.012^{a}	1.081 ± 0.102^{b}
rpotmp-2	0.174 ± 0.019^{c*}	0.123 ± 0.018^{b}	0.173 ± 0.004^{c}	0.164 ± 0.027^{c}	0.182 ± 0.019^{c}	0.216 ± 0.011^{c}	0.109 ± 0.012^{b}	0.065 ± 0.008^{a}	0.173 ± 0.014^{c}
rrn26									
WT	1.000 ± 0.133^{b}	1.110 ± 0.121^{b}	1.361 ± 0.142^{b}	0.956 ± 0.088^{b}	1.188 ± 0.122^{b}	2.114 ± 0.319^{c}	1.049 ± 0.101^{b}	0.225 ± 0.020^{a}	0.842 ± 0.103^{b}
rpotmp-2	4.312 ± 0.511^{d*}	3.110 ± 0.325^{c}	2.980 ± 0.204^{c}	3.669 ± 0.421^{c}	3.329 ± 0.325^{c}	7.559 ± 0.811^{e}	0.979 ± 0.101^{a}	1.907 ± 0.185^{b}	4.496 ± 0.447^{d}
rrn4									
WT	1.000 ± 0.101^{b}	1.974 ± 0.214^{c}	2.541 ± 0.311^{c}	1.702 ± 0.102^{c}	1.107 ± 0.099^{b}	0.901 ± 0.088^{b}	0.614 ± 0.055^{a}	0.517 ± 0.054^{a}	0.989 ± 0.099^{b}
rpotmp-2	0.913 ± 0.087^{b}	0.712 ± 0.068^{b}	0.811 ± 0.063^{b}	0.815 ± 0.091^{b}	0.804 ± 0.075^{b}	0.831 ± 0.107^{b}	0.479 ± 0.051^{a}	0.498 ± 0.012^{a}	0.810 ± 0.074^{b}
mTERF15									
WT	1.000 ± 0.098^{c}	1.678 ± 0.168^{d}	5.966 ± 0.601^{e}	2.357 ± 0.213^{d}	1.755 ± 0.134^{d}	3.009 ± 0.288^{d}	0.551 ± 0.060^{b}	0.245 ± 0.022^{a}	0.442 ± 0.039^{b}
rpotmp-2	0.485 ± 0.057^{c}	0.856 ± 0.107^{d}	2.002 ± 0.176^{e}	1.022 ± 0.099^{d}	0.610 ± 0.063^{cd}	0.904 ± 0.087^{d}	0.202 ± 0.018^{b}	0.131 ± 0.010^{a}	0.300 ± 0.034^{b}
mTERF17									
WT	1.000 ± 0.120^{d}	2.427 ± 0.255^{e}	6.277 ± 0.581^{f}	2.066 ± 0.204^{e}	2.184 ± 0.191^{e}	0.713 ± 0.069^{c}	0.503 ± 0.048^{c}	0.184 ± 0.021^{a}	0.321 ± 0.035^{b}
rpotmp-2	0.526 ±	0.511 ±	1.148 ±	0.809 ±	0.912 ±	0.395 ±	0.305 ±	0.222 ±	0.328 ±

	0.053^{c}	0.049^{c}	0.131^{d}	0.087^{d}	0.085^{d}	0.040^{b}	0.040^{b}	0.025^{a}	0.036^{b}
mTERF18									
WT	1.000 ± 0.087^{c}	1.596 ± 0.161^{d}	3.171 ± 0.320^{e}	1.821 ± 0.199^{d}	1.331 ± 0.128^{cd}	2.158 ± 0.209^{de}	0.851 ± 0.069^{bc}	0.236 ± 0.031^{a}	0.684 ± 0.066^{b}
rpotmp-2	1.213 ± 0.113^{c}	2.425 ± 0.251^{e}	2.990 ± 0.266^{e}	1.777 ± 0.164^{d}	1.796 ± 0.149^{d}	1.676 ± 0.157^{cd}	0.329 ± 0.044^{b}	0.159 ± 0.017^{a}	0.859 ± 0.073^{c}
At2g37320									
WT	1.000 ± 0.144^{c}	1.404 ± 0.161^{c}	6.649 ± 0.320^{e}	2.894 ± 0.186^{d}	2.168 ± 0.175^{d}	3.322 ± 0.360^{d}	0.374 ± 0.029^{b}	0.148 ± 0.011^{a}	0.552 ± 0.049^{b}
rpotmp-2	0.738 ± 0.063^{c}	1.417 ± 0.150^{d}	3.611 ± 0.369^{e}	1.319 ± 0.164^{d}	1.837 ± 0.171^{d}	1.681 ± 0.160^{d}	0.090 ± 0.006^{a}	0.084 ± 0.009^{a}	0.359 ± 0.034^{b}
At1g09190									
WT	1.000 ± 0.108^{c}	1.790 ± 0.139^{d}	3.017 ± 0.287^{e}	1.825 ± 0.175^{d}	1.316 ± 0.120^{d}	2.863 ± 0.293^{e}	0.841 ± 0.067^{bc}	0.230 ± 0.020^{a}	0.537 ± 0.055^{b}
rpotmp-2	1.396 ± 0.142^{d}	2.004 ± 0.196^{de}	2.671 ± 0.282^{e}	1.769 ± 0.169^{d}	1.788 ± 0.155^{d}	1.720 ± 0.163^{d}	0.356 ± 0.042^{b}	0.140 ± 0.013^{a}	0.710 ± 0.088^{c}
SWIB5									
WT	1.000 ± 0.123^{c}	3.142 ± 0.307^{d}	9.815 ± 1.005^{e}	6.386 ± 0.701^{d}	5.809 ± 0.602^{d}	6.225 ± 0.519^{d}	0.675 ± 0.074^{bc}	0.311 ± 0.029^{a}	0.502 ± 0.058^{b}
rpotmp-2	1.444 ± 0.148^{c}	2.993 ± 0.311^{d}	6.190 ± 0.717^{e}	5.364 ± 0.549^{e}	6.191 ± 0.593^{e}	3.410 ± 0.299^{d}	0.602 ± 0.059^{b}	0.376 ± 0.040^{a}	0.666 ± 0.074^{b}

Table S3. Effect of CK treatment on relative gene expression values of mitochondrial and nuclear genes. Seeds of *A. thaliana* wild-type were germinated in the dark for 4 days on full MS medium with or without cytokinin (1 µM CK). Total RNAs were isolated from seedlings and analyzed by relative quantitative RT-PCR using *UBQ10* and *PP2A* as internal standards. The data presented in the table are the mean values ($n \geq 3$). Error bars represent SEs. Different letters denote statistically significant differences between variants within the same genotype at $p < 0.05$ (ANOVA with post hoc Tukey's multiple-comparison test), asterisks indicate statistically significant differences between the control variants of mutants and the wild type at $p < 0.05$ (t-test).

**Gene**	**MS**	**1µM CK**
RPOTm	1.000±0.019^{a}	1.041±0.209^{a}
RPOTmp	1.000±0.190^{a}	0.084±0.012^{b}
nad3	1.000±0.145	0.213±0.023^{b}
nad6	1.000±0.158^{a}	0.529±0.090^{b}
cob	1.000±0.217^{a}	0.366±0.039^{b}
cox1	1.000±0.152^{a}	0.119±0.026^{b}
rps12	1.000±0.169^{a}	1.894±0.270^{b}
rrn26	1.000±0.209^{a}	0.544±0.108^{b}
atp6-1	1.000±0.228^{a}	0.397±0.055^{b}
mTERF15	1.000±0.104^{a}	0.622±0.225^{b}
mTERF18	1.000±0.090^{a}	0.446±0.043^{b}

At1g09190	1.000±0.234[a]	0.374±0.091[b]
At2g37320	1.000±0.035[a]	0.483±0.032[b]
SWIB5	1.000±0.121[a]	0.622±0.132[b]

Table S4. Putative *cis*-regulatory elements for MYB and MY-related identified in the 1,200 bp Arabidopsis *RPOTmp* promoter sequence relative to ATG *in silico* (based on the results of analysis by PlantRegMap/PlantTFDB v5.0 accessed on 10 October 2023).

Motif	Family	Seq ID	Position	Strand	Matched sequence
AT3G47600	MYB	At5g15700	503-521	-	CGACTAGTGGTTGGGTTGC
AT2G02820	MYB	At5g15700	6-20	+	cctaaacgctccatt
AT1G08810	MYB	At5g15700	503-521	+	gcaacccaaccactagtcg
AT5G62470	MYB	At5g15700	497-514	-	TGGTTGGGTTGCTAGTTG
AT3G23250	MYB	At5g15700	1148-1168	-	AACCAGAACAATAACCAACTC
AT1G66370	MYB	At5g15700	1148-1158	+	gagttggttat
AT3G28910	MYB	At5g15700	505-519	+	aacccaaccactagt
AT5G02320	MYB	At5g15700	845-859	+	gactttgggccgtta
AT2G32460	MYB	At5g15700	1148-1162	-	AACAATAACCAACTC
AT5G11510	MYB	At5g15700	846-860	+	actttgggccgttat
AT5G40330	MYB	At5g15700	275-285	-	AAGCTTGTTGC
AT1G74650	MYB	At5g15700	468-482	-	TAACTCACACCTGAT
AT5G62470	MYB	At5g15700	465-482	+	ataatcaggtgtgagtta
AT5G06100	MYB	At5g15700	1148-1158	-	ATAACCAACTC
AT4G32730	MYB	At5g15700	846-860	+	actttgggccgttat
AT1G09540	MYB	At5g15700	507-521	+	cccaaccactagtcg
AT5G02840	MYB_related	At5g15700	926-935	+	agatattttt
AT4G01280	MYB_related	At5g15700	926-936	+	agatatttttta
AT1G18330	MYB_related	At5g15700	924-935	-	AAAAATATCTAC
AT5G52660	MYB_related	At5g15700	926-936	+	agatatttttta
AT3G09600	MYB_related	At5g15700	926-937	+	agatattttttaa
AT1G01060	MYB_related	At5g15700	926-935	+	agatattttt
AT5G17300	MYB_related	At5g15700	924-937	-	TTAAAAATATCTAC
AT5G67580	MYB_related	At5g15700	915-933	-	AAATATCTACCCTAAATTC
AT1G18960	MYB_related	At5g15700	697-718	-	CCAATTAATCAACAACAAAAAA
AT3G10113	MYB_related	At5g15700	924-938	-	ATTAAAAATATCTAC
AT2G46830	MYB_related	At5g15700	926-933	-	AAATATCT

Table S5. Primers for plasmid constructions, sequencing and qRT-PCR

Name/ Purpose	Primer Name	Primer sequence* (5'-> 3')
p958/cloning P958::GUS	15700-958 Bam HI	ATT GGA TCC ATC AGT AAT TAG TAA TTT ACC TCA
cloning *Pro::GUS* general primer	15700-R1 Pst I	AAT CTG CAG AAT CGA ACT TAA AAA TTG AG
GUS/PCR and qRT-PCR	*GUS*_F	CCT CGC ATT ACC CTT ACG CT
	*GUS*_R	AAG ACA GCT GAA AGC CGA CA

RPOTm/qRT-PCR	*RPOTm*_F	ATA TGC CAA ACT CAT GCT CG
	*RPOTm*_R	CTG TTT GAC CAG CTT TCT GTC
RPOTmp/qRT-PCR	*RPOTmp*_F	CGT TTC CTC ATT TAG ACT TTC CTC C
	*RPOTmp*_R	CCT TCT CTC TGT CTG CGT CTC TGT
nad6/qRT-PCR	*nad6*_F	TAT GCC GGA AAG GTA CGA AG
	*nad6*_R	GTG AGT GGG TCA GTC GTC CT
nad3/qRT-PCR	*nad3*_F	ACC TTT TTC TTT CCT TGG GCA GT
	*nad3*_R	CGA AGC ACC CCT TTT CCA TTC A
cob/qRT-PCR	*cob* _F	TGC CGC AAT GGT ATT TCC TA
	cob _R	GCC AAA AGC AAC CAA AAC AT
cox1/qRT-PCR	*cox1*_F	AGT CAT TCT GGA GGA GCA GTT GA
	*cox1*_R	ACG GAC CAC ACA AAT AGG GGT AA
atp6-1/qRT-PCR	*atp6-1*_F	TCT TTT GCG AGT CAA TGC AC
	*atp6-1*_R	TCT CGC GTA TCT CAC ATT GC
ccMc/qRT-PCR	*ccMc* _F	AGC TAC GCG CAA ATT CTC AT
	ccMc _R	GCC GTG GCG ATA TAA ACA AT
ccMF/qRT-PCR	*ccMF* _F	CAC ATG GAG GAG TGT GCA TC
	ccMF _R	GTG GGT CCA TGT AAA TGA TCG
rps4/qRT-PCR	*rps4*_F	ACC CAT CAC AGA GAT GCA CA
	*rps4*_R	TCA CAC AAA CCC TTC GAT GA
mttB/qRT-PCR	*mttB* _F	GGG GTC TTT CTT TGG AAA CC
	mttB _R	TCT CCC TCA TTC CAC TCG TC
matR/qRT-PCR	*matR* _F	AAT TTT TGC GAG AGC TGG AA
	matR _R	TTG AAC CCC GTC CTG TAG AC
rrn26/qRT-PCR	*rrn26*_F	GAC GAG ACT TTC GCC TTT TG
	*rrn26*_R	CTT GGA GCG AAT TGG ATG AT
mTERF15/qRT-PCR	*mTERF15*_F	TCG TGC TGG GTT TGA AGT GAA
	*mTERF15*_R	ACC CTC GGC TCT TTC CAT AC
mTERF17/qRT-PCR	*mTERF17*_F	TCT TTA GTT GCC GTC TTG TCC T
	*mTERF17*_R	TCT ATC GCG GGT TTG ATC TT
mTERF18/qRT-PCR	*mTERF18*_F	GAG TGC CGT CGA AAT ACA AGT
	*mTERF18*_R	GGT GGT GTG GAG ATA GTC TGT A
At2g37320/qRT-PCR	*At2g37320*_F	AAG CGG AAC AAA GCC AGA TG
	*At2g37320*_R	CCG TGT TCC GCC ATC AAA T
At1g09190/qRT-PCR	*At1g09190*_F	ACA GGT CAG GTG GAG AGA GGA GA
	*At1g09190*_R	CGT AAT GCT CGG TTC TCG CCT
SWIB5/qRT-PCR	*SWIB5*_F	ATG GCG GGA ATT TCT AGG GT
	*SWIB5*_R	GCT ACA GTA ACG GCC TCA ACA
IAA19/qRT-PCR	*IAA19*_F	ACG AAG ACA AAG ATG GAG ACT GGA
	*IAA19*_R	GTC ATC ATC ACT CGT CTA CTC CTC T
DWF4/qRT-PCR	*DWF4*_F	GTT GGC CAT TTC TTG GTG AAA
	*DWF4*_R	TGG CGG TGT ACG GTT TAA GAT
ERF-1/qRT-PCR	*ERF-1*_F	ATT AGG GTT TGG CTC GGG AC
	*ERF-1*_R	GAC TCT TGA ACT CTC TCC GCC G
GA3/qRT-PCR	*GA3*_F	AGTCTCTTCGGGCTACCTGT
	*GA3*_R	CGGAAATCGTTGAGAGGCG
RD29/qRT-PCR	*RD29*_F	AGG AAC CAC CAC TCA ACA CAC
	*RD29*_R	GCT CGT CAT CAT CAT CAT CTT C

ARR5/qRT-PCR	*ARR5*_F	CTA CTC GCA GCT AAA ACG C
	*ARR5*_R	GCC GAA AGA ATC AGG ACA
PR1/qRT-PCR	*PR1*_F	TTC ACA ACC AGG CAC GAG GA
	*PR1*_R	CCA GCG TAG TTG TAG TTA GCC TTC
UBQ10/qRT-PCR	*UBQ10*_F	GCG TCT TCG TGG TGG TTT CTA A
	*UBQ10*_R	GAA AGA GAT AAC AGG AAC GGA AAC A

*Sequences for restriction sites Bam HI and Pst I are underlined.

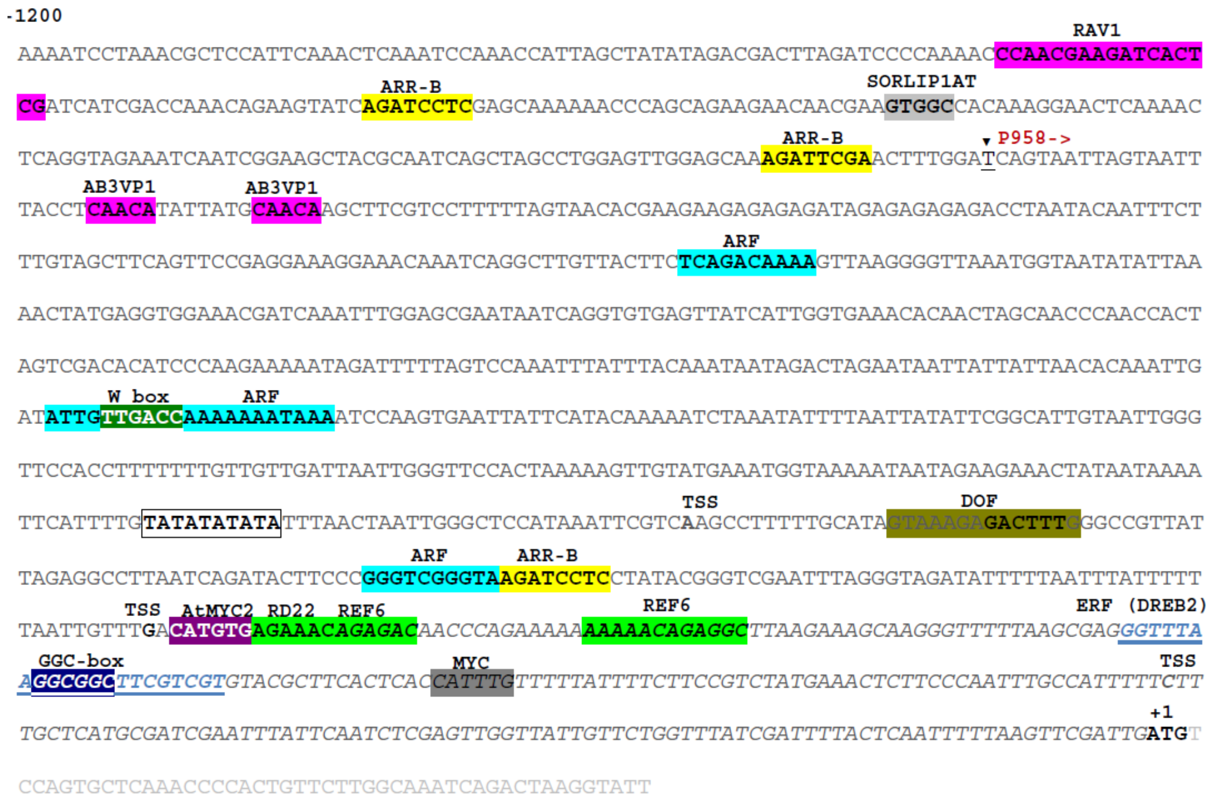

Figure S1. Nucleotide sequence (−1200/+ 50 bp) of the *RpoTmp* gene from *Arabidopsis thaliana* with distribution of *cis*-regulatory elements located with PLAN, AGRIS, PLACE, PlantTFDB v5.0. The motifs for CREs (see Table 1) were searched in the 1200 bp upstream of the translation initiation codon (bold). The 5'-UTR sequence (for *At5g15700.1* transcript) is shown in italics and marked with grey. Partial coding sequence is shown in grey letters (+50 bp). TATA boxes indicated in the frame and transcriptional start sites (TSS) printed in bold red letters were determined using the TSSPlant tool PlantProm DB on SoftBerry platform (https://www.softberry.com/ (accessed on 10 October 2023)). Significant motifs with the highest nucleotide similarity to previously identified transcription factor binding sites are shown in bold and the name of the corresponding transcription factor is given above the motif. The start position of the 5’-deletion fragment P958 is indicated by an arrow relative to ATG.

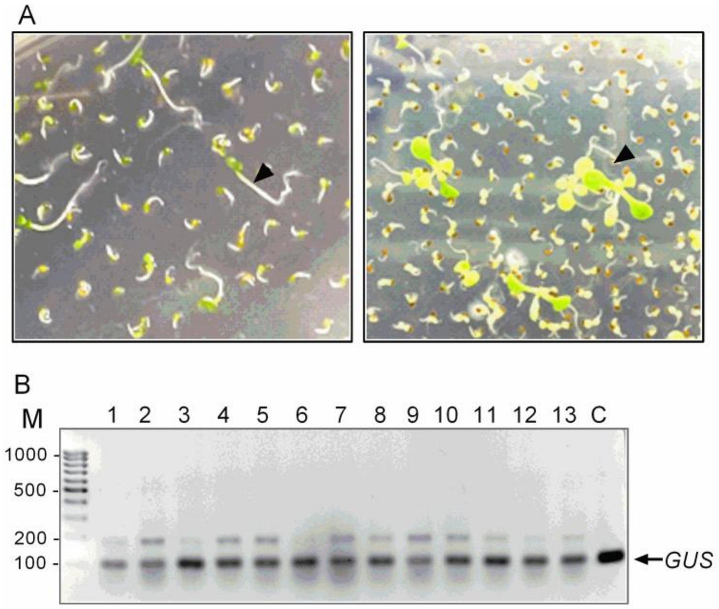

Figure S2. Generation of *RPOTmp pro::GUS* transgenic Arabidopsis plants. (A) Selection of T-seedlings carrying the P958 constructs on ½MS medium containing 0.8% (w/v) phyto agar (Duchefa Biochemie, Haarlem, The Netherlands) and 30 mg/L hygromycin (Hyg). Hyg-resistant seedlings (left photo) had more elongated hypocotyl after 3 days of growth in darkness compared to untransformed plants. Phenotype of 10-days-old transgenic seedlings (right panel) used for further analysis. (B) PCR analysis of genomic DNA extracted from rosette leaves of P958 transgenic plants using *GUS*-specific primers (Table S1), where C is the control for PCR with pCambia-35SGUS plasmid. The position of the *GUS* gene fragment (100 bp) is indicated by an arrow. M – 100 bp DNA marker (SybEnzyme, Moscow, Russia).
